# Efficacy and safety analysis of PD-1 inhibitors combined with chemoradiotherapy in the treatment of locally advanced nasopharyngeal carcinoma

**DOI:** 10.3389/fimmu.2026.1876101

**Published:** 2026-07-28

**Authors:** Lizhen Huang, Yuanqing Li, Weimei Huang, Shibin Liao, Lulu Huang, Tingting Zhang, Rensheng Wang

**Affiliations:** 1 Department of Radiation Oncology, The First Affiliated Hospital of Guangxi Medical University, Nanning, Guangxi, China; 2 Department of Oncology, The Tenth Affiliated Hospital of Guangxi Medical University (The First People’s Hospital of Qinzhou), Qinzhou, Guangxi, China; 3 Guangxi Key Laboratory of Immunology and Metabolism for Liver Diseases, Nanning, Guangxi, China; 4 Key Laboratory of Early Prevention and Treatment for Regional High-Frequency Tumor (Guangxi Medical University), Ministry of Education, Nanning, Guangxi, China

**Keywords:** chemoradiotherapy, immunotherapy, induction chemotherapy, intensity-modulated radiation therapy, locally advanced nasopharyngeal carcinoma, PD-1 inhibitors

## Abstract

**Objective:**

This study aimed to assess the efficacy and safety of integrating programmed death-1 (PD-1) inhibitors with chemoradiotherapy (CRT) in the treatment of locally advanced nasopharyngeal carcinoma (LA-NPC), explore the optimal sequencing of immunotherapy, and to compare the short-term efficacy of different combination regimens during the induction phase.

**Methods:**

We retrospectively analyzed 512 LA-NPC patients (stage III-IVA, excluding T3N0). Among these patients, 256 received PD-1 inhibitors combined with CRT, and another 256 underwent CRT alone. The two groups were matched for T stage, N stage, overall tumor stage, sex, and age. We compared outcomes including progression-free survival (PFS), distant metastasis-free survival (DMFS), locoregional failure-free survival (LRFFS), overall survival (OS), and adverse events between the two groups. Further subgroup analyses examined the 3-year PFS rates according to the timing of PD-1 inhibitor administration (induction, concurrent, and maintenance immunotherapy, induction plus concurrent immunotherapy, and induction-phase immunotherapy). We also compared the short-term efficacy of PD-1 inhibitors combined with induction chemotherapy (IC) versus IC alone, and the efficacy variations associated with antiangiogenic or anti- epidermal growth factor receptor (EGFR) therapy, different PD-1 inhibitors, and chemotherapy protocols.

**Results:**

The PD-1 inhibitor combined with CRT group showed significantly improved 3-, 4-, and 5-year rates of PFS, OS, and DMFS compared to the CRT group (3-year PFS: 90.6% vs. 82.6%, 4-year PFS: 89.3% vs. 77.3%, 5-year PFS: 89.3% vs. 76.1%; all *p* < 0.05). No notable difference was observed in LRFFS. Subgroup analyses revealed no significant variation in 3-year PFS rates among different PD-1 inhibitor administration schedules. Following IC, the PD-1 inhibitor plus IC cohort demonstrated higher complete response rates and objective response rates for primary nasopharyngeal lesions, cervical lymph nodes, and retropharyngeal lymph nodes than the IC cohort. The addition of antiangiogenic or anti-EGFR therapies further improved these response rates. The incidence of adverse events was comparable between the two groups.

**Conclusions:**

Combining PD-1 inhibitors with CRT significantly improves PFS, DMFS and OS in LA-NPC patients, with a favorable safety profile. No significant differences in efficacy were found across different PD-1 inhibitors and chemotherapy regimens, suggesting flexibility in treatment selection based on clinical circumstances.

## Introduction

Nasopharyngeal carcinoma (NPC), which originates from the nasopharyngeal mucosal epithelium, is an aggressive malignancy characterized by rapid local invasion and early metastatic potential (1). Approximately 75% of NPC patients initially present with locally advanced nasopharyngeal carcinoma (LA-NPC) (2). Currently, the therapeutic standard for LA-NPC typically involves induction chemotherapy (IC) followed by chemoradiotherapy (CRT) (3). Although local control exceeding 90% can be achieved using this combined modality, distant metastasis remains a significant issue, causing treatment failure in approximately 20%-30% of patients (4). Consequently, there is a pressing need for optimized systemic treatments aimed at decreasing the risk of distant metastasis, highlighting the urgent need for novel agents to enhance treatment effectiveness in LA-NPC.

Recently, programmed death-1 (PD-1) inhibitors have significantly reshaped cancer therapy, leading to notable improvements in clinical outcomes (5). Tumors with high programmed death-ligand 1 (PD-L1) expression and abundant immune cell infiltration usually show better responses to PD-1 inhibition. Immunohistochemical studies have indicated that NPC tissues generally express high levels of PD-L1 and exhibit substantial lymphocyte infiltration (6), supporting the rationale for PD-1 inhibitor use in NPC clinical treatment.

Previous studies demonstrated improved progression-free survival (PFS) and overall survival (OS) when PD-1 inhibitors were combined with chemotherapy as first-line treatment for recurrent or metastatic NPC. For instance, the JUPITER-02 trial (7) found that toripalimab combined with gemcitabine and cisplatin (GP) significantly enhanced PFS and OS (hazard ratio [HR] = 0.63) compared to GP alone. Similar results were observed in the CAPTAIN-1st trial (camrelizumab) (8) and the RATIONALE-309 trial (tislelizumab) (9).

The CSCO guidelines recommend PD-1 inhibitors for first-, second-, and later-line treatment in metastatic NPC. Clinical trials involving LA-NPC have demonstrated significant efficacy when PD-1 inhibitors were used as induction, concurrent, or adjuvant immunotherapy. In a study conducted by Wu et al. (10), the efficacy of an induction immunochemotherapy protocol, involving three cycles of camrelizumab combined with albumin-bound paclitaxel, cisplatin, and S-1, was evaluated using complete response (CR) as the primary endpoint. Their findings indicated a CR rate of 41.4% among patients completing all three cycles of IC, achieving an objective response rate (ORR) of 100% (10). These CR outcomes were markedly superior to those previously documented for conventional IC regimens (2.1%-11.3%) (11–13). In another randomized controlled study by Mai Haiqiang et al. (14), the efficacy of combining induction and adjuvant immunotherapy was investigated. Patients were initially treated with two cycles of IC with either toripalimab or placebo, subsequently received concurrent CRT, and then underwent an additional eight cycles of adjuvant treatment with either toripalimab or placebo. The 2-year PFS was notably higher in patients receiving toripalimab, and significant improvements in 3-year OS were also demonstrated. Moreover, the 3-year cumulative incidence of locoregional recurrence in the toripalimab group was 3.7%, while no notable differences in adverse events (AEs) were reported between the two groups. The CONTINUUM study (2) employed a comprehensive immunotherapy approach, administering a PD-1 inhibitor throughout the induction, concurrent, and adjuvant phases. Patients were randomly assigned to the control group (IC with GP followed by concurrent CRT with cisplatin alone) or to the experimental group (including sintilimab for 12 cycles). The experimental arm exhibited significantly improved 36-month event-free survival (*p* = 0.019), distant metastasis-free survival (DMFS, *p* = 0.041), and locoregional recurrence-free survival (LRFFS) (*p* = 0.043).

Although immunotherapy has shown promising clinical outcomes, the optimal timing for incorporating immunotherapy into chemotherapy or radiotherapy and the optimal choice of chemotherapy agents for LA-NPC remain uncertain and require further investigation.

This retrospective study included 512 patients with LA-NPC. Its objectives were to evaluate the efficacy and safety of PD-1 inhibitors combined with CRT in LA-NPC treatment, to explore the optimal sequencing of immunotherapy, and to compare the short-term efficacy of various combined regimens during IC. The study also aims to provide real-world evidence to enhance personalized clinical treatment strategies, thereby improving local control rates and survival outcomes for LA-NPC patients.

## Methods

### Patient selection

Clinical information for individuals diagnosed with LA-NPC (clinical stage III-IVA, excluding stage T3N0) was retrospectively gathered at the First Affiliated Hospital of Guangxi Medical University from 2020 to 2023. The study comprised 256 patients who underwent treatment involving PD-1 inhibitors combined with CRT (PD-1 plus CRT group), another 256 patients receiving CRT alone (CRT group) were matched through 1:1 propensity score matching (PSM) according to tumor stage (T and N categories), overall stage, age, and sex.

**Figure 1:**
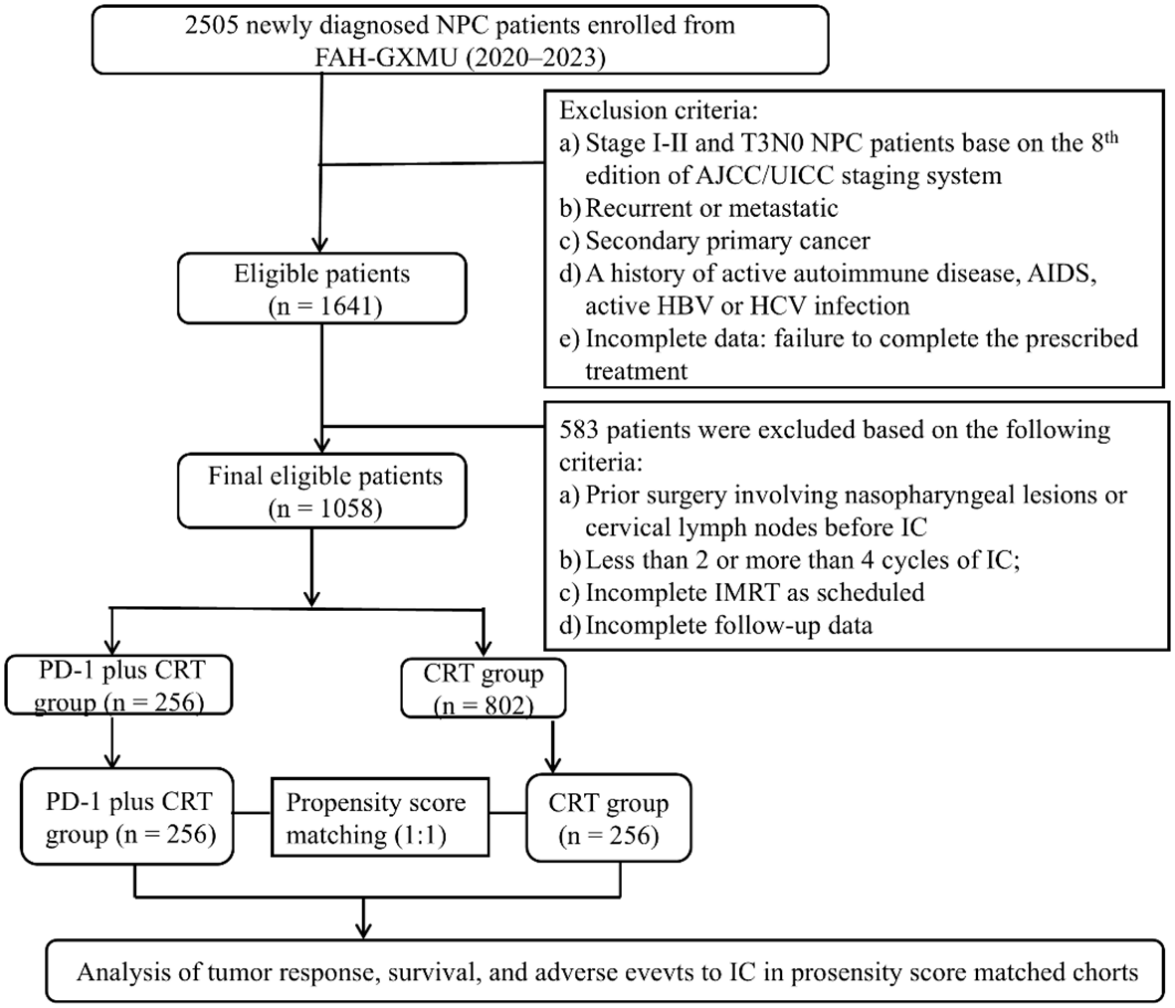
Flow chart of participant inclusion. IC, induction chemotherapy; CRT, chemoradiotherapy; FAH-GXMU, The First Affiliated Hospital of Guangxi Medical University; AIDS, acquired immunodeficiency syndrome; HBV, hepatitis B virus; HCV, hepatitis C virus; IMRT, intensity- modulated radiation therapy.

Inclusion criteria were as follows: (1) histologically confirmed NPC diagnosis; (2) age ≥18 years with an ECOG performance status ranging between 0 and 1; (3) clinical stage including III-IVA according to the 8th edition of the AJCC/UICC staging system; (4) at least one measurable lesion identified at the primary nasopharyngeal site; (5) receipt of definitive IC plus concurrent CRT or radiotherapy; (6) intensity-modulated radiation therapy (IMRT) as the standard treatment approach; (7) normal hematologic, renal, and hepatic function prior to the initiation of treatment. Exclusion criteria included: (1) incomplete medical documentation; (2) the presence of an additional primary malignancy; (3) prior surgical interventions targeting cervical lymph nodes(CLNs) or nasopharyngeal lesions before IC (4) fewer than two or more than four cycles of IC administered; (5) a history of or current autoimmune disorders, acquired immunodeficiency syndrome (AIDS), active tuberculosis, or active hepatitis B (HBV) or hepatitis C virus (HCV) infections; and (6) incomplete IMRT or inadequate follow-up information. The flowchart for excluding and including patients is shown in **Figure 1**.

### Chemotherapy, PD-1 inhibitors, and anti-angiogenic/anti- epidermal growth factor receptor therapeutic agents

All patients received 2 to 4 cycles of IC containing platinum before IMRT. Regimens consisted of taxane plus platinum, including albumin-bound paclitaxel, paclitaxel liposome, or docetaxel combined with cisplatin or nedaplatin. The GP regimen included gemcitabine with cisplatin at the same dose as above. All chemotherapy regimens were administered intravenously and repeated every three weeks. PD-1 inhibitors used included camrelizumab, toripalimab, sintilimab, or tislelizumab intravenously on day 1 of each IC cycle. In patients who also received PD-1 inhibitors during CRT and as maintenance therapy after radiotherapy, the dosage and administration schedule matched those used during the IC phase. Anti-angiogenic and anti-EGFR therapies involved recombinant human endostatin and nimotuzumab.

### Radiotherapy

After IC, all patients received definitive IMRT five fractions per week. The prescribed doses for IMRT were as follows: nasopharyngeal gross tumor volume (GTVnx), 68.8–72 Gy in 32–34 fractions; gross tumor volume of metastatic cervical lymph nodes (GTVnd), 64-70.62 Gy in 32–34 fractions; clinical target volume 1 (CTV1, high-risk subclinical disease), 60–66 Gy in 32–34 fractions; and clinical target volume 2 (CTV2, low-risk subclinical disease), 54–56 Gy in 32–33 fractions. During radiotherapy, patients received one of the following concurrent treatment regimens: single-agent chemotherapy; chemotherapy combined with nimotuzumab; or chemotherapy combined with anti-angiogenic therapy.

### Treatment response

The primary outcomes included PFS, DMFS, LRFFS, and OS among patients receiving PD-1 inhibitor combined with CRT. Secondary outcomes comprised a comparison of 3-year PFS rates among patients receiving induction, concurrent, and maintenance immunotherapy, induction plus concurrent immunotherapy, or induction-phase immunotherapy alone. Subgroup analyses were performed to compare the survival benefits of PD-1 inhibitor treatment, including PFS, OS, DMFS, and LRFFS, between patients with elevated baseline EBV DNA levels and those with normal baseline EBV DNA levels. Additional endpoints included CR and ORR after IC, the CR rate at one-year post-radiotherapy, and safety profiles associated with immunotherapy. Imaging modalities, including computed tomography (CT) of the cervical area and nasopharynx if magnetic resonance imaging (MRI) was contraindicated, along with CT assessments of the abdomen and chest, were utilized to evaluate short-term therapeutic effectiveness. Short-term responses were classified following Response Evaluation Criteria in Solid Tumors (RECIST, version 1.1) into CR, partial response (PR), stable disease (SD), or progressive disease (PD). The ORR combined CR and PR rates. All outcomes, including safety data, were reviewed and verified by attending physicians. Patients exhibiting residual or recurrent malignancies post-treatment received subsequent therapeutic interventions as clinically indicated, including chemotherapy, additional radiotherapy, surgical management, targeted therapy, or immunotherapy.

### Assessment of treatment toxicity

Comprehensive laboratory tests were conducted to evaluate toxicity. Additionally, AEs such as nausea and gastrointestinal symptoms were monitored. All AEs were assessed according to the Common Terminology Criteria for AEs (CTCAE) version 5.0.

### Survival and prognostic factors

The PFS rate was defined as the proportion of patients without disease progression or death from any cause at a given follow-up point. The DMFS rate represented the percentage of patients free from distant metastasis and alive at a specific time point. LRFFS indicated the proportion of patients free from recurrence in the local or regional area at a designated follow-up period. OS represented the proportion of patients surviving at specific follow-up intervals. Kaplan-Meier methods were employed to estimate survival probabilities for PFS and OS, with log-rank tests used for analyzing survival differences across three separate patient groups. Median survival durations and corresponding 95% confidence interval (CI) were calculated from the survival curves.

### Follow-up

Follow-up evaluations were performed every 3 months within the initial two years after treatment, subsequently at 6-month intervals from years three to five, and annually thereafter until patient death. Comprehensive follow-up assessments included: (1) physical examinations and nasopharyngoscopy; (2) imaging with nasopharyngeal and cervical MRI or CT; (3) routine blood analysis, including serum tumor markers; (4) quantitative measurement of EBV DNA levels. Medical records were systematically reviewed throughout the treatment period and during follow-up to ensure accurate data collection. Survival status was confirmed through medical records and telephone interviews, with a final data cutoff date of November 30, 2025. For all time-to-event analyses, survival intervals were calculated from the date of initial diagnosis until event occurrence or the most recent follow-up.

### Statistical analysis

Baseline characteristics of patients were assessed using matched chi-square analyses. Continuous variables were compared using Student’s t-tests, while categorical variables and efficacy indicators (ORR and CR rates) were compared between treatment groups through chi-square analyses. AEs were summarized as frequencies and percentages. Differences in PFS, DMFS, OS, and LRFFS were analyzed using Kaplan-Meier curves. SPSS software version 25.0 was applied for statistical analyses, and a *p* value less than 0.05 was considered statistically significant.

## Results

### Baseline characteristics of patients

A total of 512 patients diagnosed with LA-NPC satisfied the inclusion criteria for further analysis (**Figure 1**), the baseline characteristics before patient matching are detailed in **Supplementary Table 1**. Patients were grouped into two cohorts based on their therapeutic regimens: a group treated with PD-1 inhibitors combined with CRT (PD-1 plus CRT group) and a group receiving CRT exclusively (CRT group). Comparisons of baseline clinical parameters revealed no significant differences between the two cohorts (all *p* > 0.05, **Table 1**).

**Table 1:** Baseline clinical characteristics of LA-NPC patients between two groups.

Baseline Characteristics	PD-1 Plus CRT Group n (%)	CRT Group n (%)	Total (n=512) n (%)	*p*
Gender Male Female	189 (73.8) 67 (26.2)	198 (77.3) 58 (22.7)	387 (75.6) 125 (24.4)	0.354
Age(years)				0.362
≤50	164 (64.1)	154 (60.2)	318 (62.1)	
>50	92 (35.9)	102 (39.8)	194(37.9)	
ECOG				0.354
0	243 (94.9)	238 (93)	481 (93.9)	
1	13 (5.1)	18 (7.0)	31 (6.1)	
AJCC Stage				0.686
III	30 (11.7)	33 (12.9)	63 (12.3)	
IVA	226 (88.3)	223 (87.1)	449 (87.7)	
T				0.667
1	0 (0)	1 (0.4)	1 (0.2)	
2	4 (1.6)	3 (1.2)	7 (1.4)	
3	77 (30.1)	75 (29.3)	152 (29.7)	
4	175 (68.4)	177 (69.1)	352 (68.8)	
N				0.752
0	5 (2)	8 (3.1)	13 (2.5)	
1	58 (22.7)	52 (20.3)	110 (21.5)	
2	71 (27.7)	76 (29.7)	147 (28.7)	
3	122 (47.7)	120 (46.9)	242 (47.3)	
Baseline EBV DNA Status				0.926
normal	168 (65.6)	169 (66)	337 (65.8)	
elevated	88 (34.4)	87 (34)	175 (34.2)	
BMI				0.124
≤18.5	23 (9)	32 (12.5)	55 (10.7)	
18.5-24	164 (64.1)	142 (55.5)	306 (59.8)	
>24	69 (27)	82 (32)	151 (29.5)	

BMI, Body mass index; ECOG, Eastern Cooperative Oncology; N, lymph node; T, tumor; AJCC, American Joint Committee on Cancer; EBV-DNA (Epstein Barr virus-deoxyribonucleic acid. ≥ 4000 copies/mL) was elevated.

### PFS, DMFS, LRFFS, and OS

**Figure 2:**
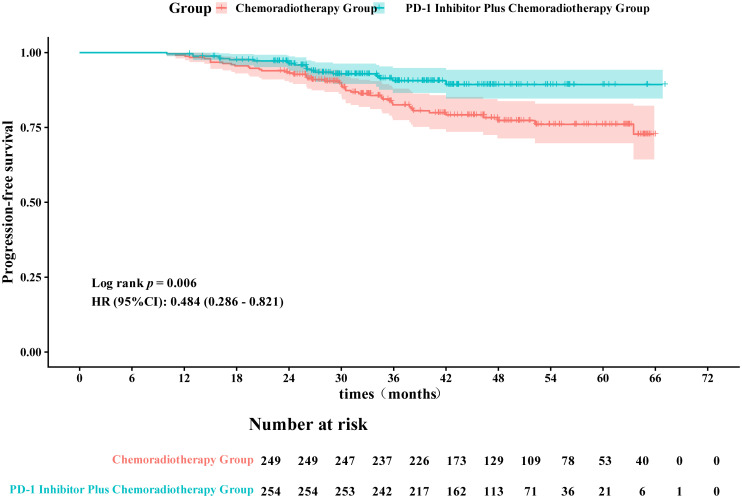
Comparison of progression-free survival between the two groups.

**Figure 3:**
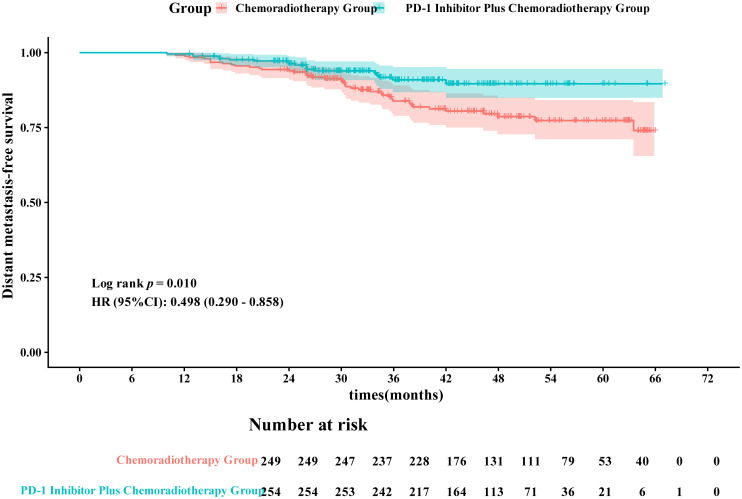
Comparison of distant metastasis-free survival between the two groups.

**Figure 4:**
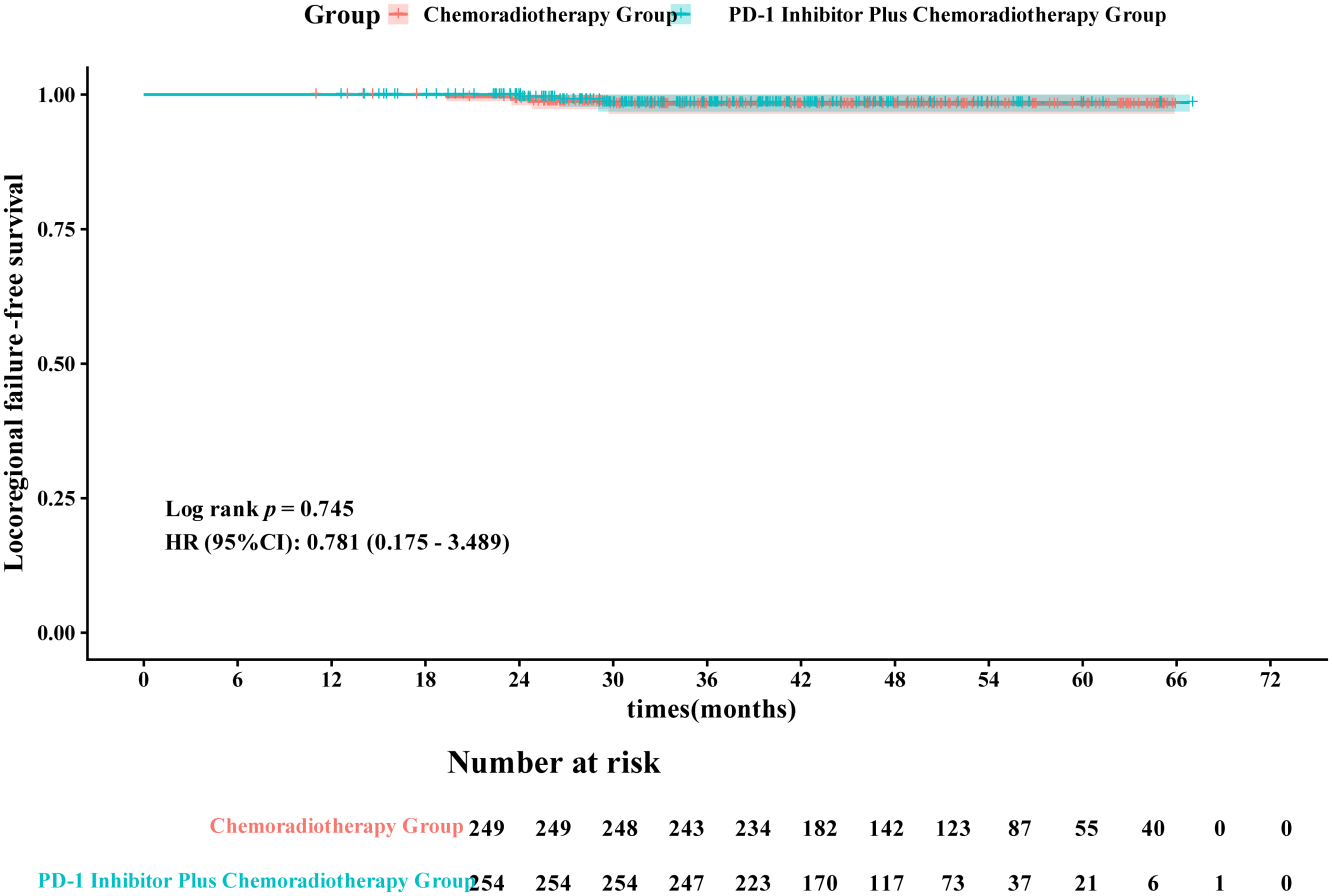
Comparison of locoregional failure-free survival between the two groups.

**Figure 5:**
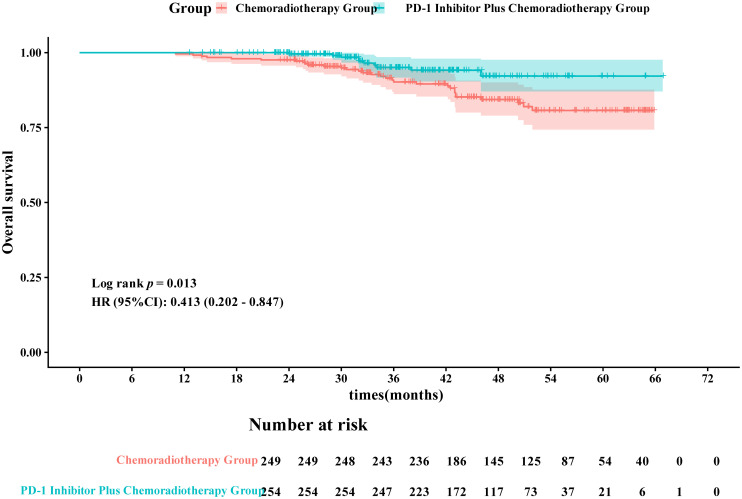
Comparison of overall survival between the two groups.

Several patients were lost to long-term follow-up, reducing the number of valid cases included in the final statistical analysis. Ultimately, 254 patients in the PD-1 plus CRT group and 249 patients in the CRT group were included in the final analysis. The PFS was notably prolonged in the PD-1 plus CRT group (mean ± SD: 62.57 ± 0.92 months) compared to the CRT group (*p* = 0.006; **Figure 2**). Similarly, DMFS was significantly improved in the PD-1 plus CRT group (*p* = 0.010; **Figure 3**). However, LRFFS between the two cohorts showed no statistically significant difference (*p* = 0.745; **Figure 4**). Additionally, OS was superior in the PD-1 plus CRT group than the CRT group (*p* = 0.013; **Figure 5**). The median duration of follow-up was 39.33 months, ranging from 11.00 to 66.83 months. Collectively, the PD-1 plus CRT cohort demonstrated enhanced 3- to 5-year rates for PFS, DMFS, and OS compared to the CRT cohort (**Tables 2**–**4**). Conversely, LRFFS rates at 2- to 5-year intervals did not differ significantly between the groups (**Table 5**).

**Table 2:** PFS rates in the PD-1 plus CRT and CRT groups.

Therapeutic effect	PD-1 plus CRT group (n=254)	CRT group (n=249)	*p*
2-year PFS rate	0.963	0.931	0.096
3-year PFS rate	0.906	0.826	0.004
4-year PFS rate	0.893	0.773	<0.001
5-year PFS rate	0.893	0.761	<0.001

**Table 3:** DMFS rates in the PD-1 plus CRT and CRT groups.

Therapeutic effect	PD-1 plus CRT group (n=254)	CRT group (n=249)	*p*
2-year DMFS rate	0.963	0.939	0.192
3-year DMFS rate	0.909	0.839	0.007
4-year DMFS rate	0.896	0.786	0.002
5-year DMFS rate	0.896	0.774	0.001

**Table 4:** OS rates in the PD-1 plus CRT and CRT groups.

Therapeutic effect	PD-1 plus CRT group (n=254)	CRT group (n=249)	*p*
2-year OS rate	0.996	0.976	0.054
3-year OS rate	0.950	0.902	0.004
4-year OS rate	0.922	0.843	<0.001
5-year OS rate	0.922	0.807	<0.001

**Table 5:** LRFFS rates in the PD-1 plus CRT and CRT group.

Therapeutic effect	PD-1 plus CRT group (n=254)	CRT group (n=249)	*p*
2-year LRFFS rate	0.996	0.992	0.551
3-year LRFFS rate	0.985	0.982	0.684
4-year LRFFS rate	0.985	0.982	0.684
5-year LRFFS rate	0.985	0.982	0.684

### Comparison of 3-Year PFS rates across different immunotherapy approaches

**Figure 6:**
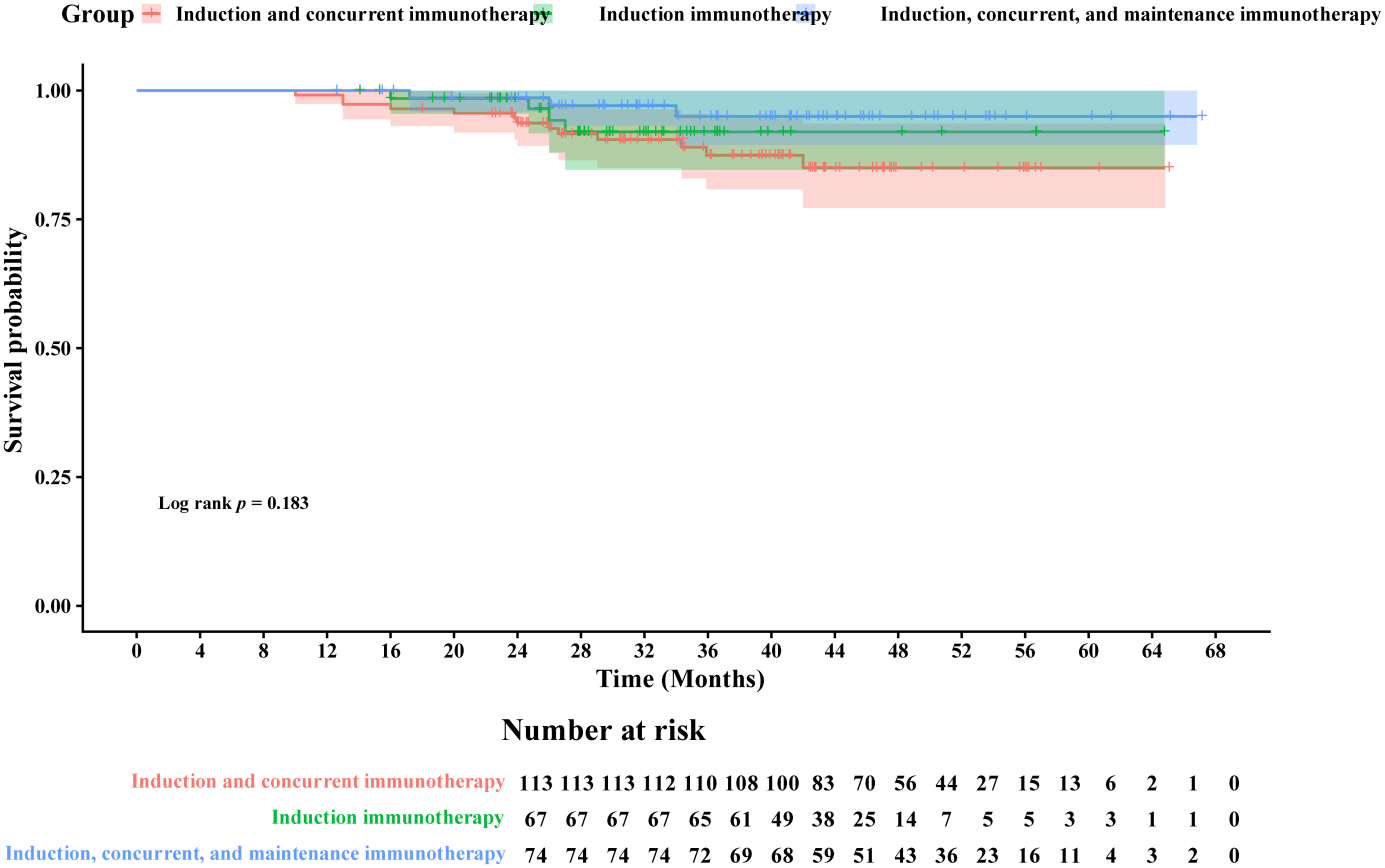
Comparison of 3-year progression-free survival rates among the induction, concurrent, and maintenance immunotherapy, induction plus concurrent immunotherapy, and induction phase immunotherapy groups.

Patients were classified into three immunotherapy regimen groups. The 3-year PFS rates were 95.0% for induction, concurrent, and maintenance immunotherapy group, 87.4% for induction plus concurrent immunotherapy group, and 92.0% for induction-phase immunotherapy group. Differences across these immunotherapy modalities were not statistically significant (*p* = 0.183, **Figure 6**).

### Association between baseline EBV DNA levels and survival outcomes

**Figure 7:**
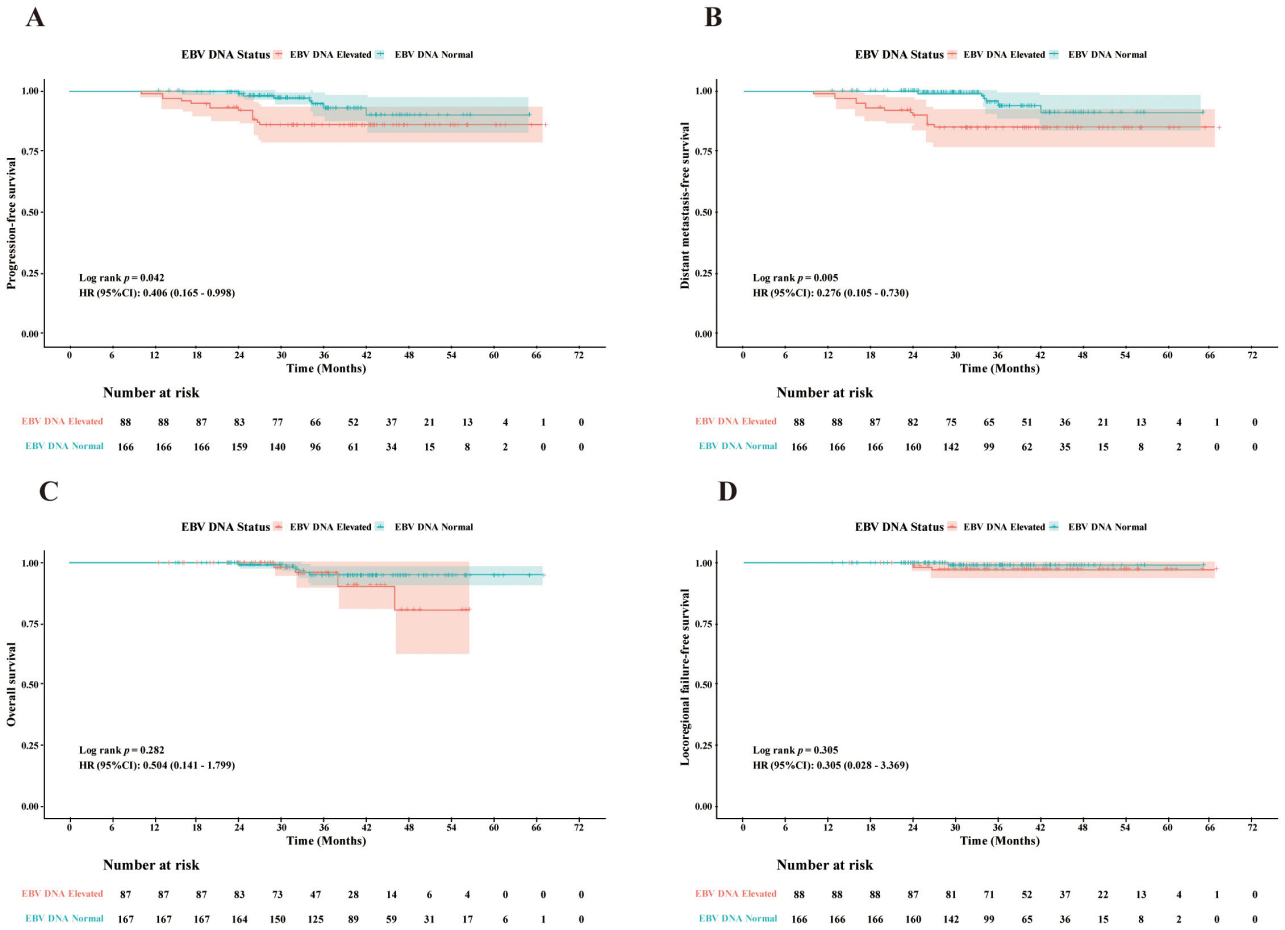
Comparison of **(A)** progression-free survival, **(B)** distant metastasis-free survival, **(C)** overall survival, **(D)** locoregional failure-free survival between patients with elevated and normal baseline EBV DNA levels.

In the PD-1 plus CRT group, NPC patients with normal EBV DNA levels had significantly longer PFS than those with elevated EBV DNA levels (mean ± SD: 61.71 ± 1.09 vs. 60.27 ± 1.77 months; *p* = 0.042; **Figure 7A**). Consistent with this finding, DMFS was also superior in the normal EBV DNA group (mean ± SD: 62.24 ± 1.04 vs. 59.66 ± 1.84 months; *p* = 0.005; **Figure 7B**). In contrast, no significant differences were observed between the normal and elevated EBV DNA groups in OS (mean ± SD: 65.01 ± 0.72 vs. 53.43 ± 1.46 months; *p* = 0.282; **Figure 7C**) or LRFFS (mean ± SD: 65.80 ± 0.73 vs. 64.50 ± 0.32 months; *p* = 0.305; **Figure 7D**).

### Effect of induction immunochemotherapy on short-term efficacy

Evaluation of response rates post-IC therapy revealed superior treatment outcomes in the PD-1 plus IC group compared to the IC group (*p* < 0.05). Specifically, the PD-1 plus IC cohort exhibited total tumor burden (including primary nasopharyngeal tumors, CLNs, and retropharyngeal lymph nodes (RPLNs)) CR in 60 patients, PR in 166, SD in 30, and no cases of PD, corresponding an ORR of 226. In contrast, the IC group had 22 patients achieving CR, 135 PR, 98 SD, and 1 PD, totaling an ORR of 157.

Further analysis explored efficacy separately for primary nasopharyngeal tumors, CLNs, and RPLNs. The addition of PD-1 inhibitors to IC significantly enhanced the CR, ORR, and SD rates for all three lesion sites (*p* < 0.05). However, PD incidence did not differ significantly (*p* = 1). PR rates showed no statistically significant difference for primary tumors and CLNs (*p ≥* 0.05), whereas significantly higher PR rates were observed for RPLNs in the PD-1 inhibitor plus IC cohort (**Table 6**).

**Table 6:** Comparative efficacy of induction PD-1 plus IC group versus IC alone group.

Lesion site	Therapeutic effect	PD-1 plus IC (n, %) (n=256)	IC (n, %) (n=256)	χ² value	*p*
Total tumor burden	CR	60(23.4)	22(8.6)	20.97	<0.001
	PR	166(64.8)	135(52.7)	7.747	0.005
	SD	30(11.7)	98(38.3)	48.17	<0.001
	PD	0(0)	1(0.4)		1
	ORR	226(88.3)	157(61.3)	49.34	<0.001
Primary nasopharynx lesions	CR	111(43.4)	56(21.9)	26.88	<0.001
	PR	109(42.6)	102(39.8)	0.395	0.53
	SD	36(14.1)	97(37.9)	37.79	<0.001
	ORR	220(85.9)	158(61.7)	38.86	<0.001
	PD	0(0)	1(0.4)		1
CLNs	CR	103(40.2)	61(23.8)	15.82	<0.001
	PR	116(45.3)	133(52)	2.260	0.133
	SD	37(14.5)	61(23.8)	7.269	0.007
	ORR	219(85.5)	194(75.8)	7.826	0.005
	PD	0(0)	1(0.4)		1
RPLNs	CR	167(65.2)	92(35.9)	43.95	<0.001
	PR	61(23.8)	92(35.9)	8.958	0.003
	SD	28(10.9)	70(27.3)	22.26	<0.001
	ORR	228(89.1)	184(71.9)	24.06	<0.001
	PD	0(0)	2(0.8)		0.499

At the one-year post-radiotherapy evaluation among patients who subsequently underwent concurrent CRT, 11 patients in the PD-1 plus IC cohort and 24 in the IC cohort missed the scheduled follow-up and were excluded, leaving 245 and 232 evaluable patients, respectively. Our results revealed CR in 231 patients in the PD-1 plus IC cohort compared to 214 patients in the IC only cohort, however, this difference was not statistically significant (*p* = 0.372, **Table 7**).

**Table 7:** One-year post-radiotherapy efficacy: induction PD-1 plus IC versus IC alone.

Therapeutic effect	PD-1 plus IC (n, %) (n=245)	IC (n, %) (n=232)	χ² value	*p*
CR (1 year After Radiotherapy)	231(94.3)	214(92.2)	0.796	0.372

11 patients in the PD-1 plus IC cohort and 24 in the IC cohort missed the scheduled follow-up and were excluded, leaving 245 and 232 evaluable patients.

### Impact of different immunotherapy drugs on post-induction efficacy

In total, 175 patients received immunotherapy with PD-1 inhibitors, including toripalimab (n=93), sintilimab (n=36), tislelizumab (n=40), and camrelizumab (n=6). The therapeutic effectiveness of two chemotherapy regimens (gemcitabine-platinum and taxane-platinum) in combination with each PD-1 inhibitor was systematically assessed. The analysis revealed no statistically significant differences in clinical efficacy among the different PD-1 inhibitors (further details are provided in the **Supplementary Table 2**). Likewise, comparisons of therapeutic responses across chemotherapy regimens, when paired with identical immunotherapy agents, also showed no significant differences (further details are provided in the **Supplementary Table 3**).

### Impact of combined medication during induction immunotherapy on treatment efficacy

In addition, the efficacy of combining PD-1 inhibitors with anti-angiogenic or anti-EGFR agents plus chemotherapy was compared to PD-1 inhibitors plus chemotherapy during induction treatment. Among 81 patients treated with PD-1 inhibitors in combination with anti-angiogenic or anti-EGFR agents, 26 achieved CR, and 76 reached ORR. Among the 175 patients treated with PD-1 inhibitors combined with chemotherapy, CR was reported in 34 patients, with an ORR of 150 patients. A statistically significant difference emerged in CR rates between the groups (*p* = 0.026), nevertheless, no significant differences were observed regarding ORR or CR rates at 3 months post-radiotherapy (all *p*>0.05) (**Table 8**).

**Table 8:** Efficacy comparison of induction therapy using PD-1 plus anti-angiogenic/anti-EGFR plus chemotherapy versus PD-1 plus chemotherapy.

Therapeutic effect	Group A (n, %) (n=81)	Group B (n, %) (n=175)	*p*
CR after IC	26(32.1)	34(19.4)	0.026
ORR after IC	76(93.8)	150(85.7)	0.061
CR rate at 3 months after IMRT	68(87.2)	140(86.4)	0.268

Group A= PD-1 plus anti-angiogenic/anti-EGFR plus Chemotherapy; Group B= PD-1 plus Chemotherapy; 3 patients in Group A and 13 in Group B missed the 3-month post-IMRT CR assessment, resulting in 78 and 162 evaluable patients for analysis.

### Adverse events

Regarding safety, the incidence of treatment-related AEs was compared between patients who received PD-1 inhibitors combined with CRT and those who underwent CRT alone. The most frequently observed grade 1–2 AEs included hematologic toxicities, electrolyte disturbances, hypoproteinemia, and elevated levels of indirect bilirubin. The most frequent grade 3–4 AEs was hematological toxicity, primarily neutropenia, followed by electrolyte disturbances, there was no significant difference in the incidence of these events between the two groups. No instances of grade 5 AEs resulting in mortality were documented (**Table 9**).

**Table 9:** Comparison of adverse events between PD-1 plus CRT versus CRT group.

AEs	PD-1 plus CRT group G1-G2 (n, %)	CRT group G1-G2 (n, %)	*p*	PD-1 plus CRT group G3-G4 (n, %)	CRT group G3-G4 (n, %)	*p*
Leukopenia	147(57.4)	141(55)	0.593	32(12.5)	19(7.4)	0.055
Anemia	221(86.3)	227(88.7)	0.423	32(12.5)	28(10.9)	0.583
Thrombocytopenia	42(16.4)	28(10.9)	0.072	10(3.9)	4(1.6)	0.104
Neutropenia	123(48)	120(46.8)	0.791	129(50.4)	139(54.3)	0.376
AST↑	64(25)	55(21.5)	0.346	4(1.6)	5(1.9)	0.737
ALT↑	68(26.5)	66(25.7)	0.841	6(2.3)	6(2.3)	1.000
Total bilirubin↑	24(9.3)	28(10.9)	0.558	2(0.7)	2(0.7)	1.000
Indirect bilirubi↑	152(59.4)	149(58.2)	0.788	2(0.7)	4(1.6)	0.411
Direct bilirubin↑	41(16.0)	7(14.5%)	0.623	2(0.7)	2(0.7)	1.000
Hypoproteinemia	200(78.1)	199(77.7)	0.915	0(0)	0(0)	–
LDH ↑	8(3.1)	10(3.9)	0.631	2(0.7)	0(0)	0.156
CK ↑	11(4.3)	4(1.56)	0.067	2(0.7%)	2(0.7)	1.000
CK-MB↑	5(1.95)	6(2.34)	0.761	0(0)	0(0)	–
AP ↑	3(1.17)	4(1.6)	0.704	1(0.3)	0(0)	0.317
Creatinin↑	101(39.4)	97(37.8)	0.717	0(0)	1(0.3)	0.317
Hypokalemia	232(90.6)	237(92.6)	0.426	13(5.1)	14(5.5)	0.843
Hyponatremia	189(73.8)	178(69.5)	0.281	2(0.7)	2(0.7)	1.000
Hypocalcemia	111(43.3)	101(39.4)	0.370	4(1.6)	3(1.2)	0.704
Nausea and vomiting	46(17.9)	49(19.1)	0.733	0(0)	0(0)	–
Fatigue	18(7)	16(6.25)	0.723	0(0)	0(0)	–
Rash	1(0.3)	0(0)	0.317	0(0)	0(0)	–
RCCEP	1(0.3)	0(0)	0.317	0(0)	0(0)	–
Hypothyroidism	27(10.5)	33(12.9)	0.410	0(0)	0(0)	–
Hyperthyroidism	55(21.4)	39(15.2)	0.068	0(0)	0(0)	–

AEs, adverse events; CRT, chemoradiotherapy; ALT, alanine aminotransferase; AST, aspartate aminotransferase; CK, Creatine Kinase; CK-MB, Creatine Kinase-Myocardial Band; AP, Alkaline phosphatase; RCCEP, Reactive capillary endothelial proliferation; LDH, Lactate dehydrogenase.

## Discussion

This study chiefly assessed the safety and effectiveness of integrating PD-1 inhibitors with CRT in patients diagnosed with LA-NPC. The outcomes indicated substantially prolonged median PFS and OS in the PD-1 plus CRT group (*p* = 0.006 and 0.013, respectively). Specifically, the 3-, 4-, and 5-year PFS rates were significantly higher in the PD-1 plus CRT group at 90.6%, 89.3%, and 89.3% than in the control cohort at 82.6%, 77.3%, and 76.1% (*p* = 0.004, < 0.001, and < 0.001, respectively). Likewise, notable statistical differences were observed in OS rates at 3-, 4-, and 5-year follow-ups (*p* < 0.05). These results suggest *p*otential enhancements in long-term survival when PD-1 inhibitors are added to traditional CRT. Although previous studies have yielded varied outcomes, for example, one earlier clinical investigation (14) evaluated toripalimab administration during induction and adjuvant phases reported a higher 3-year OS rate (99% vs. 90%) in the experimental group, though it failed to reach statistical significance. Such outcomes might be influenced by shorter follow-up durations, subsequent therapeutic interventions, or limited participant numbers, necessitating longer observation periods for validation. The results showed longer median DMFS and LRFFS in the PD-1 plus CRT group. The difference was statistically significant for DMFS (*p* = 0.010) but not for LRFFS (*p* = 0.745). The DMFS findings were consistent with those of a previous prospective study (14), whereas the LRFFS results were inconsistent.

Chemotherapy influences the host anti-tumor immune response through various mechanisms, including promoting tumor antigen presentation, altering the expression patterns of immune regulatory receptors and their ligands, modifying cytokine secretion, and activating innate immune pathways. These actions establish an immunological environment favorable for subsequent immunotherapy (15). Radiotherapy enhances the effectiveness of immunotherapy by inducing immunogenic cell death, releasing tumor-associated antigens that facilitate better antigen presentation and processing. Furthermore, radiotherapy-induced DNA damage activates the cGAS-STING pathway, which subsequently elevates type I interferon production, attracts immune cells to the tumor region, and establishes an inflammatory tumor microenvironment (TME) conducive to improved immune responses. The subsequent activation of T cells post-radiotherapy boosts cytokine secretion, notably interferon-γ, significantly raising PD-L1 expression in both tumor and immune cells. This creates a distinct target for PD-1 inhibitors (16). Therefore, combining PD-1 inhibitors with CRT results in synergistic anti-tumor activity, producing complementary and amplified immunotherapeutic effects.

In the subgroup comparison based on the administration timing of PD-1 inhibitors, the induction, concurrent, and maintenance immunotherapy group showed a numerically superior 3-year PFS rate (95%) than the induction-only group (92%) or induction plus concurrent immunotherapy group (87.4%), however, these differences lacked statistical significance. Such outcomes may stem from relatively small and uneven subgroup sizes as well as limited follow-up intervals, necessitating validation through expanded studies with longer observation periods. The use of PD-1 inhibitors after CRT may sustain host anti-tumor immune responses, continuously eliminate minimal residual disease, and reduce recurrence and metastasis risk. These findings align with results from the CONTINUUM study (2), which evaluated sintilimab as part of induction, concurrent, and maintenance immunotherapy for LA-NPC, and another trial (14) evaluating toripalimab in induction and adjuvant settings for LA-NPC. Despite limited continuation of immunotherapy in the induction-phase-only group, the 3-year PFS rate remained high at 92%, highlighting the “tail-of-the-curve” effect of immunotherapy and emphasizing the importance of early initiation. However, the optimal timing of immunotherapy requires confirmation through prospective studies.

The relatively lower PFS observed in patients receiving induction plus concurrent immunotherapy might be attributed to radiotherapy-induced lymphocyte depletion. Conventional fractionated radiotherapy, typically delivered at daily doses of 1.8–2.0 Gy, repeatedly exposes radiosensitive T-cell populations, potentially diminishing subsequent immunotherapeutic responses (15, 16). Extensive irradiation fields may further directly reduce memory T-cell numbers, thereby compromising immunotherapy benefits (17). In support of this possibility, a clinical trial (18) involving locally advanced head and neck squamous cell carcinoma patients without molecular subtype screening found no survival advantage when pembrolizumab was added to CRT, in fact, CRT alone achieved superior event-free survival. Similar conclusions have been drawn from additional clinical studies (19–21). Thus, the optimal integration of radiotherapy and immunotherapy remains unresolved due to complexities surrounding immunotherapy timing and radiation fractionation schedules. Currently, no head-to-head trials definitively identify the most effective combination approach.

In the PD-1 plus CRT group in this study, NPC patients with high EBV DNA levels had poorer PFS and DMFS than those with low EBV DNA levels, whereas no significant differences in OS or LRFFS were observed between the two subgroups. Plasma EBV DNA load is well recognized as an important tumor biomarker in NPC and is widely used for screening, monitoring, and prognostic prediction. A previous meta-analysis showed that pretreatment EBV DNA levels can effectively predict OS, PFS, DMFS, and LRFFS in patients with NPC (22). In a cohort of patients with recurrent or metastatic NPC treated with PD-1 inhibitors, those with high baseline EBV DNA levels had a lower durable clinical benefit rate than those with low baseline EBV DNA levels (19.6% [19/97] vs. 38.0% [27/71], *p* = 0.01) (23). Patients with low baseline plasma EBV DNA levels derived significantly greater PFS benefit than those with high EBV DNA levels (HR = 0.52, 95% CI: 0.42-0.63, *p*<0.001). In contrast, no significant difference in PFS was observed between patients with decreased and increased EBV DNA loads during immunotherapy (HR = 0.51, 95% CI: 0.22-1.17, *p* = 0.109) (24), suggesting that baseline EBV DNA levels may have a greater prognostic impact than dynamic changes during treatment. Plasma EBV-encoded latent membrane proteins can target and degrade interferon receptors, suppress interferon-mediated antiviral and antitumor responses, and thereby enable NPC cells to evade host immune surveillance (25). EBV noncoding RNAs mediate immune evasion in NPC cells via suppression of tumor antigen presentation and immune cell activation (26, 27). Elevated EBV DNA levels may therefore reflect a higher burden of tumor cells that can evade immune elimination, ultimately contributing to disease progression and poor clinical prognosis.

In assessments concentrating on the induction phase, patients receiving PD-1 inhibitors alongside chemotherapy exhibited notably higher rates of CR and ORR compared to chemotherapy alone, with marked improvements observed in primary nasopharyngeal tumors, CLNs, and RPLNs. Particularly impressive were the CR (65.2%) and ORR (86%) rates for RPLNs, exceeding those previously documented (28). However, at one year after radiotherapy, there was no significant difference in CR rates between the two groups, suggesting that the primary benefit of adding PD-1 inhibitors during IC is a reduction in tumor stage. While CRT remains the cornerstone treatment for LA-NPC, immunotherapy serves as a crucial adjunctive approach. Despite similar one-year CR rates, notable disparities existed in long-term DMFS. This difference likely reflects the primary role of PD-1 inhibitors in achieving deeper remission and eliminating circulating micro metastases rather than amplifying local radiosensitivity.

Furthermore, subgroup analysis investigating induction-phase therapeutic combinations revealed that patients receiving PD-1 inhibitors plus either anti-angiogenic or anti-EGFR targeted agents and chemotherapy may achieve higher CR rates than patients treated only with PD-1 inhibitors plus chemotherapy (*p* = 0.026). Nimotuzumab, an EGFR-specific monoclonal antibody, inhibits EGFR-driven signaling pathways, effectively reducing angiogenesis. High EGFR expression occurs in over 70% of NPC patients and correlates with poorer clinical outcomes (29, 30). Consistently, a recent study (31) found a significantly improved 3-year PFS rate in the immunotherapy + nimotuzumab + chemotherapy group (96.6%, 95% CI: 93.2%–100%) compared with the nimotuzumab + chemotherapy group (79.8%, 95% CI: 75.6%–84%, *p* = 0.04), accompanied by an acceptable safety profile (only 5.3% grade ≥ 3 AEs). EGFR-targeting monoclonal antibodies may also alter the TME, augment tumor immunogenicity, and facilitate anti-tumor immune responses.

The anti-angiogenic agent employed was recombinant human endostatin (Endostar). Endostar targets vascular endothelial growth factor receptors to inhibit tumor angiogenesis (32). At doses promoting vascular normalization, it can alleviate immunosuppression in the TME, polarize tumor-associated macrophages, enhance T-cell infiltration, and improve immunotherapy efficacy. In another study (33), the combination of Endostar, PD-1 inhibitors, and chemotherapy was evaluated as first-line treatment for advanced non-small cell lung cancer (NSCLC) without EGFR or anaplastic lymphoma kinase mutations. This combination yielded a notable ORR (72.06%), high disease control rate (95.59%), median PFS of 22.0 months (95% CI: 16.6-27.4), and median OS of 31.0 months (95% CI: 23.4-NE). These real-world studies have provided preliminary validation of these findings, suggesting that the addition of anti-angiogenic or anti-EGFR agents may confer favorable survival benefits with a manageable safety profile.

Additionally, research (34) involving multitargeted anti-angiogenic tyrosine kinase inhibitors combined with PD-1 inhibitors and chemotherapy as second-line treatment in recurrent or metastatic NPC revealed significantly prolonged PFS (*p* < 0.001) and OS (*p* = 0.042), an increased ORR (*p* = 0.041), and a trend toward enhanced disease control rates (*p* = 0.126) with only a slight, non-significant increase in AEs (*p* = 0.599). These findings underscore the therapeutic potential of anti-angiogenic agents combined with PD-1 inhibitors and chemotherapy in managing NPC. Nevertheless, the relatively small anti-angiogenic subgroup in the present study limits the robustness of these analyses. Further prospective studies with larger cohorts are warranted to validate these findings.

Moreover, comparative evaluations among different PD-1 inhibitors (toripalimab, sintilimab, tislelizumab, and camrelizumab) combined with identical chemotherapy regimens revealed no statistically significant differences in clinical efficacy. These findings may suggest comparable antitumor activity among these agents in the induction treatment of LA-NPC. Similarly, a real-world study (35) involving Chinese patients with NSCLC who received immune checkpoint inhibitors (pembrolizumab, camrelizumab, tislelizumab, sintilimab, and nivolumab) found no notable differences in efficacy or safety, which may offer preliminary support for individualized PD-1 inhibitor selection based on patient tolerability and economic considerations. However, the relatively small sample size within each subgroup in this study may have limited the reliability of these analyses. Larger prospective studies are warranted to confirm these observations and to guide precise clinical decision-making.

From a safety perspective, the occurrence of grade 1–2 AEs, predominantly hematologic toxicities, electrolyte imbalances, hypoproteinemia, showed no significant differences between the PD-1 plus CRT and CRT groups. Although grade 3–4 neutropenia and hypocalcemia were slightly higher in the PD-1 plus CRT cohort, these differences did not achieve statistical significance, suggesting that the addition of PD-1 inhibitors does not markedly increase severe AE risk in LA-NPC patients. In terms of immune-related adverse events (irAEs), hyperthyroidism was slightly more frequent, while hypothyroidism was less common in the PD-1 inhibitor-treated patients. Overall, irAEs remained relatively uncommon, with no grade 5 treatment-related fatalities recorded.

Several limitations of this study should be recognized. Firstly, as a retrospective investigation, inherent selection biases exist, and residual confounding cannot be entirely ruled out despite statistical corrections. The present study also involved treatment heterogeneity and potential confounding factors. Multiple regimens were included in this cohort, including four types of PD-1 inhibitors, diverse IC strategies involving taxane-based and GP regimens, and additional concurrent nimotuzumab or anti-angiogenic agents during radiotherapy. Although subgroup analyses did not reveal significant prognostic differences among these treatment combinations, the inclusion of heterogeneous therapeutic regimens inevitably introduced confounding bias in this retrospective study. Importantly, the relatively small sample sizes within subgroups limit the statistical power and precision of these comparisons. Future investigations with larger cohorts and standardized treatment protocols are needed to enable robust stratification and validation of these preliminary observations. Secondly, the limited duration of follow-up precludes conclusive evidence regarding long-term survival benefit, necessitating extended patient monitoring. Thirdly, immune-related biomarkers such as tumor mutational burden and PD-L1 expression were not assessed, thereby limiting the ability to identify patients most likely to benefit from immunotherapy. Finally, due to the very small number of patients treated with PD-1 inhibitors during both induction and adjuvant phases, this subgroup was excluded from the analysis. Future prospective randomized controlled trials should incorporate biomarker analysis, optimize patient selection, and define the optimal treatment combinations, dosing, and duration, to improve individualized management strategies for LA-NPC.

## Conclusions

In summary, PD-1 inhibitors combined with CRT improved PFS, OS, and DMFS in LA-NPC patients, with a manageable safety profile. The 3-year PFS rates were 95%, 87.4%, and 92% in the induction, concurrent, and maintenance immunotherapy, induction plus concurrent immunotherapy, and induction-phase immunotherapy groups, respectively, with no statistically significant differences. Following induction therapy, the PD-1 inhibitor plus IC cohort demonstrated higher CR and ORR than the IC-only cohort. The addition of anti-angiogenic/anti-EGFR agents during induction therapy may further enhance the treatment efficacy. Different PD-1 inhibitors show comparable effectiveness and can be flexibly selected in clinical practice. These findings provide preliminary evidence to support clinical decision-making.

## Data Availability

The original contributions presented in the study are included in the article/**Supplementary Material**. Further inquiries can be directed to the corresponding authors.
