# Supplementary material for: Efficacy and safety analysis of PD-1 inhibitors combined with chemoradiotherapy in the treatment of locally advanced nasopharyngeal carcinoma
Source: Front Immunol. 2026 Jul 28;17:1876101. doi: 10.3389/fimmu.2026.1876101 (PMC13457740; doi:10.3389/fimmu.2026.1876101)
Supplement: Supplementary file 1 [file Table1.docx]

**Supplementary Table1 Pre-matching Baseline Table of Patients**

|  |  | PD-1 Plus CRT Group | CRT Group | Total (n=512) | *p* |
| --- | --- | --- | --- | --- | --- |
| Gender | male | 602（75.1%） | 198（73.8%） | 791（74.8%） | 0.692 |
|  | female | 200（24.9%） | 67（26.2%） | 267（25.2%） |  |
| Age（years） | ≤50 | 164（64.3%） | 719（89.7%） | 883（83.5%） | ＜0.001 |
|  | >50 | 91（35.7%） | 83（10.3%） | 174（16.5%） |  |
| AJCC stage | III | 30（11.7%） | 254（31.7%） | 284（26.8%） | ＜0.001 |
|  | IVA | 226（88.3%） | 548（68.3%） | 774（73.2%） |  |
|  | 1 | 0（0%） | 11（0.4%） | 11（1%） | ＜0.001 |
|  | 2 | 4（1.6%） | 44（5.5%） | 48（4.5%） |  |
| T | 3 | 77（30.1%） | 308（38.4%） | 385（36.4%） |  |
|  | 4 | 175（68.4%） | 439（54.7%） | 614（58%） |  |
|  | 0 | 5（2%） | 29（3.6%） | 34（3.2%） | ＜0.001 |
|  | 1 | 58（22.7%） | 254（31.7%） | 312（29.5%） |  |
| N | 2 | 71（27.7%） | 281（35%） | 352（33.3%） |  |
|  | 3 | 122（47.7%） | 238（29.7%） | 360（34%） |  |

**Supplementary Table2 Efficacy Evaluation of Different Immunotherapeutic Agents During Induction Therapy**

| **Chemotherapy Regimen** | **Immunotherapy A** | **Immunotherapy B** | **CR Rate after (****Immunotherapy A + IC)** | **CR Rate after (Immunotherapy B + IC)** | ***P*** |
| --- | --- | --- | --- | --- | --- |
| Gemcitabine + platinum | Toripalimab | Camrelizumab | 17（22.4%） | 1 (50.0%) | 0.36 |
|  | Toripalimab | Sintilimab | 17（22.4%） | 1 (6.7%) | 0.163 |
|  | Toripalimab | Tislelizumab | 17（22.4%） | 3 (17.6%) | 1 |
|  | Camrelizumab | Tislelizumab | 1 (50.0%) | 3 (17.6%) | 0.386 |
|  | Camrelizumab | Sintilimab | 1 (50.0%) | 3 (6.7%) | 0.228 |
|  | Sintilimab | Tislelizumab | 3 (6.7%) | 3 (17.6%) | 1 |
| Paclitaxel + platinum | Toripalimab | Camrelizumab | 3 (17.6%) | 0 (0%) | 1.000 |
|  | Toripalimab | Sintilimab | 3 (17.6%) | 9 (42.9%) | 0.096 |
|  | Toripalimab | Tislelizumab | 3 (17.6%) | 4 (17.4%) | 1 |
|  | Camrelizumab | Sintilimab | 0(0%) | 9 (42.9%) | 0.26 |
|  | Camrelizumab | Tislelizumab | 0(0%) | 4 (17.4%) | 1 |
|  | Sintilimab | Tislelizumab | 9 (42.9%) | 4 (17.4%) | 0.064 |

IC = induction chemotherapy；CR = complete response

**Supplementary Table 3 Efficacy Evaluation of Different Chemotherapy Regimens During Induction Therapy**

| Immunotherapy Agent | Chemotherapy A | Chemotherapy B | CR Rate after Induction (Immuno + Chemo A) | CR Rate after Induction (Immuno + Chemo B) | *p* |
| --- | --- | --- | --- | --- | --- |
| Toripalimab | Gemcitabine + platinum | Paclitaxel + platinum | 17(22.4%) | 2 (12.5%) | 0.509 |
| Tislelizumab | Gemcitabine + platinum | Paclitaxel + platinum | 1 (6.7%) | 5 (23.8%) | 0.367 |
| Sintilimab | Gemcitabine + platinum | Paclitaxel + platinum | 3 (17.6%) | 4 (17.4%) | 1.000 |
| Camrelizumab | Gemcitabine + platinum | Paclitaxel + platinum | 1 (50.0%) | 1 (25%) | 1.000 |

CR = complete response
